# Catalogue of type specimens of fungi and lichens deposited in the Herbarium of the University of Granada (Spain)

**DOI:** 10.3897/BDJ.3.e5204

**Published:** 2015-07-13

**Authors:** M. Teresa Vizoso, Carmen Quesada

**Affiliations:** ‡ Herbarium of University of Granada, Rector López Argüeta, 8. 18071, Granada, Spain; § Curator at Herbarium of University of Granada, Rector López Argüeta, 8. 18071, Granada, Spain

**Keywords:** Occurrence, specimen, nomenclature, type material, herbarium collection, image collection, western Mediterranean, Spain, Mycology, Fungi, Ascomycota, Basidiomycota, Glomeromycota, Lichenized Fungi, GDA, GDAC, *
Cortinarius
*

## Abstract

**Background:**

A catalogue of types from the Herbarium of the University of Granada has not previously been compiled. As a result, a search of these collections in order to compile digital images for preservation and publication yielded a large number of formerly unrecognized types.

**New information:**

This dataset contains the specimen records from the catalogue of the nomenclature types of fungi and lichens in the Herbarium of the University of Granada, Spain. These herbarium specimens are included in the GDA and GDAC collections, acronyms from Index Herbariorum (Thiers 2014). At this time, the type collection of fungi and lichens contains 88 type specimens of 49 nominal taxa, most from *Agaricales* and the genus *Cortinarius*, described from the western Mediterranean, mainly Spain, by the following authors: V.Antonin, J.Ballarà, A.Bidaud, G.F.Bills, M.Bon, C.Cano, M.Casares, G.Chevassut, M.Contu, F.Esteve-Raventós, R.Galán, L.Guzmán-Dávalos, R.Henry, E.Horak, R.Mahiques, G.Malençon, P.Moënne-Loccoz, G.Moreno, A.Ortega, F.Palazón, V.N.Suárez.-Santiago, A.Vêzda, J.Vila, and M.Villareal.

For each specimen, the locality indication, species name, observation date, collector, type status, related information, associated sequences, other catalogue numbers related to each type, and image URL are recorded. The dataset is associated with an image collection named “Colección de imágenes de los tipos nomenclaturales de hongos, líquenes, musgos y algas incluidos en el Herbario de la Universidad de Granada (GDA y GDAC)” (Vizoso and Quesada 2013) which is housed and accessible at the Global Biodiversity Information Facility in Spain (GBIF.ES) Hosting and Publishing Service “Biodiversity Image Portal of Spanish collections” and is also available at the Herbarium of University of Granada institutional web (Vizoso 2014a, Vizoso 2014b). That image collection contains 113 images, of which 56 correspond to the nomenclature types of 49 taxa (47 fungi, 2 lichens), the rest of the images in this collection correspond to documents and specimens or microscopy photographs which are included in the herbarium specimens of fungi. These complement and document the process of the typification.

## Introduction

 The Herbarium of the University of Granada combines two general collections: GDA and GDAC (Thiers 2014). The GDA was formerly the herbarium of the Pharmacy Faculty, which was created in 1852. In 1970, GDAC was created in the Science Faculty. In 2000, both herbaria were moved to the same place, forming the current Herbarium of the University of Granada. The GDAC collection was closed in 2000 with a total of 45,000 records. New materials are continuously added to the collection and registered with the GDA acronym. Currently, the GDA Herbarium has become an essential reference for studies of the flora of south-eastern Spain. Both collections, the GDA and GDAC, include specimens of vascular plants, algae, bryophytes and fungi. Vascular plants amount to 87% of the whole collection (including a 3% of pteridophytes) and the rest of the groups (algae, bryophytes, fungi and lichens) 13% (including a 9% in the GDAC and 4% in the GDA). Over 50% of all vascular-plant specimens are in the GDA collection, which continues to grow, and the rest belongs to the GDAC. Conversely, over 69% of the algae, bryophytes and fungi come from the GDAC. Within this group, the fungus (51%), lichen (25%, completely within the GDA) and moss (18%) collections have special significance. The remaining specimens correspond to algae and liverworts. Geographically, the country with the most specimens is Spain (94%), followed by Morocco (3.5%) and Portugal (1.5%). Within Spain, the largest portion is from Andalusia (82%) which in turn includes samples from Granada (68%), Almeria (11%), Jaen (7%), Malaga (6%), and other Andalusian provinces, confirming the value of our Herbarium as a reference for studying the flora from eastern Andalusia.

## General description

### Purpose

Since the unification of the GDA and GDAC collections, many tasks have been accomplished. These include a complete review of all materials, recovery and incorporation of unregistered materials, updating curatorial methods and, even more important, the computerization of the entire collection. More recently, the Herbarium of the University of Granada has developed several projects to digitalize images of high-priority specimens in order to preserve them and make them available on Internet. As a result of the reviewing process, many types that had not previously been compiled were detected. In addition, the catalogues of type specimens of different groups of the Herbarium of the University of Granada have been compiled, published, and made accessible on Internet through the “Biodiversity Image Portal of Spanish collections” at the Global Biodiversity Information Facility in Spain (GBIF.ES) Hosting and Publishing Service and at the Herbarium of the University of Granada institutional web. The catalogue of type specimens of fungi and lichens has been one of the first published on the GBIF.ES Integrated Publishing Toolkit (IPT) (GBIF.ES 2012). This dataset is the most important part of the image collection “Colección de imágenes de los tipos nomenclaturales de hongos, líquenes, musgos y algas incluidos en el Herbario de la Universidad de Granada (GDA y GDAC)” (Vizoso and Quesada 2013) published in GBIF.ES and “Tipos nomenclaturales de hongos” (Vizoso 2014a) and “Tipos nomenclaturales de líquenes” (Vizoso 2014b) available on the Herbarium of University of Granada institutional web site.

## Project description

### Title

Towards a digital image collection of the Herbarium of the University of Granada

### Personnel

M.Teresa Vizoso

### Study area description

The application of new technologies in the field of natural-history collections is enabling herbaria not only to manage their collections more effectively but also to provide access to a large volume of biodiversity information through computerization and the creation of a specimen database. After this step, databases can enrich their contents by digitizing images associated with high-priority specimens such as nomenclatural types. This represents a major advance in the preservation of sensitive materials. For example, the risk of damage involved in consulting and sending sensitive material is minimized. It is also vital in terms of enhancing information and reaching out to wider audiences at multiple levels, since, at the database level, database records can have links to the associated image (Häuser et al. 2005). At a more general level, institutional, national (e.g. GBIF.ES) or global (e.g., Encyclopedia of Life) species catalogues can combine this information with their own and provide it to multiple audiences. In this project, the GDA Herbarium has sought to respond mainly to the increasing demand for the use of the images in order to preserve sensitive material. The herbarium started to generate digital images of three of its most sensitive collections: nomenclatural types, the historical collection of Amo y Mora (1852) (the oldest collection of this herbarium) and other digital images of sheets from 200 Andalusian singular taxa that illustrated the "Singular Flora from Andalusia" species portal, also developed by the herbarium. The techniques that allow the incorporation, operation, and management of new digital-image collections, as well as those which facilitate its accessibility via Internet have also been implemented.

### Design description

The main objective was to ensure the preservation of sensitive material of the University of Granada Herbarium by implementing high-quality curatorial standards as well as to generate, manage, and disseminate the associated data in digital formats. This was achieved by:

- Creating and making available on Internet an image collection of singular taxa from Andalusia that meets the needs of researchers, managers, and the general public.
- Obtaining a detailed report on the number, location, and treatment of nomenclatural types in the University of Granada Herbarium in order to perform a database-cleaning process and to complete the curatorial management of this sensitive material.
- Compiling a collection of electronic publications (protologues) that support the study of nomenclatural types and improves accessibility and service that the University of Granada Herbarium offers to researchers.
- Ensuring the preservation of the historic collection of Amo y Mora (the oldest collection of this herbarium, started in 1852 by M. Amo y Mora) by fully digitizing it and publishing it online.

### Funding

The Herbarium of the University of Granada is part of the research group called “The Herbarium of the University of Granada as a Source for Taxonomic, Environmental and Biodiversity Studies” (group code: 288RNM) which is one the Research and Technological Development groups from Regional Government of Andalusia (Junta de Andalucía), Spain. The funds were provided by the Technical Support Program, under the Scientific-Technological Infrastructure modality of the Ministry of Science and Innovation of Spanish Goverment and the University of Granada. The project was undertaken from February 2010 to July 2013.

## Sampling methods

### Study extent

This study includes fungus and lichen collections. The fungus collection of the herbarium comes from two herbaria, one of which originated at the Pharmacy Faculty (GDA) and the other at the Science Faculty (GDAC). In the year 2000, the two herbaria were joined. The Herbarium of the University of Granada (GDA) maintains both sections and, although both collections retain their individuality, new materials are added only to the GDA collection. Currently, the overall fungus collection comprises 7244 specimens derived mostly from research conducted by Dr Antonio Ortega. Some 97% of the specimens come from Spain, followed by just under 2% that correspond mostly to exsiccate Uredineen Sydow (1874-1889) of a collection of *Uredinales*, *Ustilaginales*, and *Erysiphales*, most from Germany and a small portion from other countries such as Hungary, Austria, and Sweden. There is also a small number from Portugal and other Mediterranean countries such as France and Italy as well as minor samples from northern Morocco, Algeria, and Tunisia.

The lichen collection has 3300 specimens, which come exclusively from the herbarium of the Pharmacy Faculty (GDA). This collection started in 1980 with materials that came from the research of Dr Manuel Casares and is subsequently enriched with exsiccate exchange with other institutions. Of this collection, 93% comes from Spain, 75% of which were collected in the south-eastern provinces of Granada, Alicante, Almeria, and Jaen. Other well-represented areas include the south-western provinces of Huelva and Cadiz, central provinces of Madrid, Salamanca, and the north-eastern provinces of Tarragona and Zaragoza. We also found a small representation from Morocco (3.3%) and the rest from Austria, Brazil, Georgia, and the Czech Republic.

### Sampling description

The fungus collection has been fully computerized and approximately 50% of the lichen collection has also been. Therefore, developing a complete catalogue involved three kinds of type specimens, namely those that: 1) already have a record in the databases; 2) specimens not recorded as a type in the databases but have been identified as such in the collection; and 3) type specimens not recorded in the databases nor identified or treated as such in the collection. For the first kind, quality control was carried out (see next section). For the second kind of type specimens, the literature from the two main researchers and collectors was reviewed. This led to the identification of type specimens that were not included in the databases and were either identified as such in the collection (case 2) or had not been identified as such (case 3). The review of the literature of these authors provided new data to both the collection and the associated databases. All type specimens are now registered in the GDA-GDAC Fungus and Lichen collections and in some cases another number from other herbaria has been included in RelatedInformation or OtherCatalogNumber fields from DarwinCore standard (Wieczorek et al. 2009). This information was provided by gifts and duplicate materials from AH, F, CFB herbaria (Index Herbariorum) and personal herbaria of M. Bon (M.B.) and G. Chevassut (Chev.).

### Quality control

The consistency of data on type-specimen records was verified by comparisons with the information in original publications of the corresponding new species. When some information items such as geographical coordinates, altitude, or identifiers of genetic sequence associated data had not been included in the herbarium database and these data were available in the protologue, they were included in this dataset. The consigned data refer to the original identification for which the nomenclatural type from the taxon name was given. When the nomenclatural type was not recorded as such in the herbarium but was found after searching and reviewing the relevant literature, a revision tag was included in the specimen record. This tag specifies the type status, name, and site where it was validly published and the author who documented it. These data have also been computerized. DARWIN TEST (Ortega-Maqueda and Pando 2008) is the software application used to validate and check records from tables in a Darwincore format before exporting database to a Darwin Core Archive file. DARWIN TEST has been used to check scientific names against the Scientific_Names table from Species 2000 (Species2000 2015), to convert coordinates from UTM to decimal degrees which are used in the Darwincore format and to detect anomalous ASCII characters. Once checked and corrected, these records were exported as a Darwin Core Archive file which was uploaded to the IPT (Integrated Publishing Toolkit) hosted by GBIF.ES (GBIF.ES 2012). The metadata from the dataset have been completed directly in the IPT.

### Step description

First of all, a query was made to each of the two herbarium databases on nomenclatural types of the fungus and lichen collections. The first database (fungi) is managed with the BIOMEN software application (Delgado et al. 2005), and the second with HERBAR (Pando et al. 1994-2010). Thus the results of the queries were compared with the label information for the type specimens in the Herbarium collection. Three kinds of errors were detected and corrected: first, typographical errors; second, records erroneously described as types; and third, omissions in the database, i.e. types that should have been recorded as such. Publications on the description of new species made by Dr Ortega and Dr Casares, the main collectors and researchers of these collections, were reviewed. After this review, 5 holotypes, 1 isotype, 1 neotype, 1 epitype and 33 paratypes that had not been recorded as types nor had received the appropriate physical curation treatment were detected. After the database update with the inclusion of new types obtained from reviewed publications and data cleaning, the collection corresponding to this dataset was obtained by consulting the database again. Data resulting from this query were manually converted to DarwinCore format. Then, the resulting DarwinCore records were completed by adding the ImageURL and TypeStatus fields, after which it was validated with the DARWIN TEST tool (Ortega-Maqueda and Pando 2008). Finally the DarwinCore Archive was generated to incorporate the metadata in this file and published it on the GBIF.ES Integrated Publishing Toolkit (IPT).

In an effort to fill out the ImageURL field in the step described above, the following steps were taken: after the catalogue of types of fungi and lichens were obtained, all specimens were checked for appropriate physical curation treatment (placed in a type cover, by convention red, to indicate the presence of type specimens and to ensure better protection). Then, digital images of the nomenclatural type and documents included with each specimen were prepared. A Hewlett Packard Scan Jet 5300C model with a resolution of 600 dpi for specimens and 150 dpi for documents was used. After the metadata for each image was included, the corresponding tiled/pyramid TIFF was generated and uploaded at the GBIF.ES “Biodiversity Image Portal of Spanish collections” Hosting and Publishing Service. This collection is available both at “Colección de imágenes de los tipos nomenclaturales de hongos, líquenes, musgos y algas incluidos en el Herbario de la Universidad de Granada (GDA y GDAC)” (http://www.gbif.es/Imagenes.php#GDA-TIPOS-CRIPTO) and also at the Herbarium of University of Granada institutional web (http://herbarium.ugr.es/pages/imagenes/tipos-nomenclaturales/tipos_hongos).

## Geographic coverage

### Description

**Figure 1.**
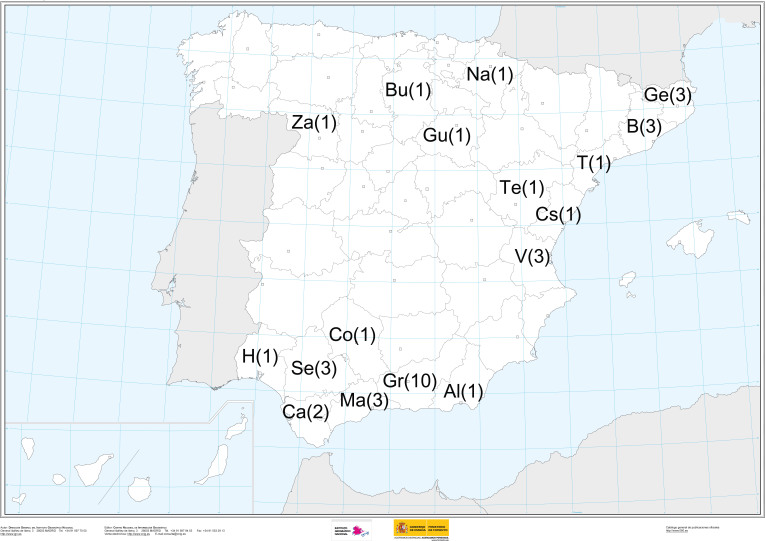
Spanish provinces with the number of taxa with type specimens in the GDA Herbarium. Source: Map was taken from National Geographic Institute (IGN, Instituto Geográfico Nacional, Gobierno de España).

In general, the distribution of the taxa in this catalogue is the western Mediterranean region. Most of the taxa are from the Iberian Peninsula (72 types records from 39 taxa) as shown in Fig. 1, smaller numbers from France (6 types from 5 taxa) and Italy (8 records from 3 taxa) and a single taxon from Morocco. Outside this general geographic distribution, there is a single taxon from Czechoslovakia with 2 records. All these type specimens from outside of Spain were gifts or exchanges from the private herbaria of Dr Ortega’s French and Italian colleagues (G.Chevassut, M.Bon, P.Möenne-Loccoz, and M.Contu). Additionally, type specimens from northern and eastern Spain come from collaboration between Dr Ortega and some researchers such as J. Vila and R. Mahiques from the Societat Catalana de Micologia and Societat Micologica Valenciana, respectively. Most of the records from Spain are from Andalusia (types of 21 taxa from 39 in Spain) and within this region the greatest portion corresponds to the provinces of Granada (10 taxa), Malaga (3), Seville (3), Cadiz (2), Cordoba (1), and Almeria (1), as shown in the Fig. 1.

### Coordinates

33°23'60'' and 50°42'0'' Latitude; 6°30'36'' and 15°26'60'' Longitude.

## Taxonomic coverage

### Description

**Figure 2.**
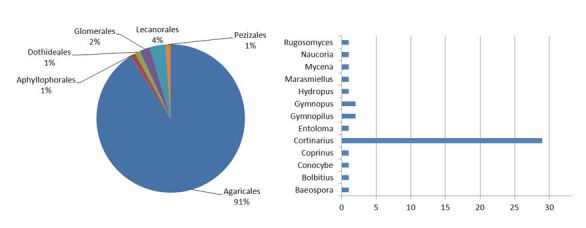
Distribution of the type specimens according to the orders in which they are included. At the right, the genera from the Agaricales order according to the number of taxa with type-specimens are represented (numbers and tables included in Suppl. material 1).

The main taxonomic coverage of this dataset corresponds to *Basidiomycota*, which constitutes 92% of the type specimens, followed by *Ascomycota* (6%, included *Lecanorales* as lichenized fungi) and a minor record of *Glomeromycota*, as shown in the Fig. 2. *Agaricales* is the most represented order (91%) and, within this order, specimens of the genus *Cortinarius* dominate the collection (56%). Fungal diversity in the Mediterranean basin is high. Populations of taxa differing morphologically with respect to their northern vicariants are frequent. Consequently, many taxonomic proposals have been made in order to explain Mediterranean fungal variability, which is common in the case of the genus *Cortinarius* (Ortega et al. 2008). This is the result of both its overall diversity and the special interest in this genus of the late Dr Antonio Ortega, the main collector and researcher in the fungus collection. As shown in the Fig. 2, *Cortinarius* is the genus with a greater number of nomenclatural types.

Note: the terms used to complete the type status reference for this dataset are from the International Code of Nomenclature for algae, fungi, and plants (ICN) (McNeill et al. 2012) and Hawksworth (2010) for the obsolete terms which are not included in the current Code. Index Fungorum (2014) has been used to verify the currently accepted names.

**Figure 3.**
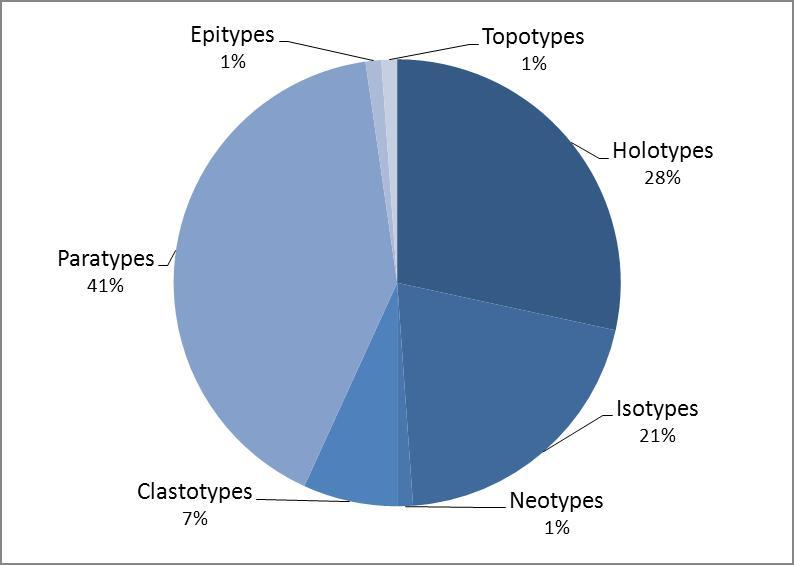
Distribution of herbarium type-specimens according to type status (Suppl. material 1).

Fig. 3 shows the range of the different type status of the catalogue. The highest number of records are paratypes (36) followed by holotypes (25), isotypes (18), clastotypes (5 fragments from holotypes, one from neotype), and a single neotype, epitype and topotype.

Of the 49 taxa, 34 are accepted in their original position (marked by an asterisk* in the taxonomic ranks) and four had already been combined into other genera: *Sarcodon mediterraneus* A.Ortega & Contu to *Beenakia*, *Rugosomyces pudicus* Bon & Contu to *Calocybe*, *Glomus custos* C. Cano & Dalpé to *Rhizophagus*, and *Bacidia subtilis* Vêzda to *Fellhanera*. One proved to be a synonym of a previously described species (Cortinarius haasii var. quercus-ilicicola A.Ortega, Suár.-Sant. & J.D.Reyes of *Cortinarius callochrous* (Pers.) Gray), another has been combined into a supraspecific rank (Gymnopus dryophilus var. lanipes (Malençon & Bertault) A.Ortega, Antonín & Esteve-Rav.), and the rest (9) have not been accepted into the proposed infraspecific rank (variety and one form): Conocybe arrhenii var. squamosipes A.Ortega & Esteve-Rav., Cortinarius assiduus var. plesiocistus A.Ortega, Vila & Bidaud, Cortinarius caesiostramineus var. cadinanos-aguirrei Moënne-Locc. & A.Ortega, Cortinarius caerulescens var. praetermissus (Bergeron ex Reumaux) A.Ortega & Moënne-Locc., Cortinarius vernus var. nevadavernus Suár.-Sant. & A.Ortega, Entoloma griseocyaneum var. glyciosmus Esteve-Rav. & A.Ortega, Hydropus floccipes var. luteipes A.Ortega & M.Zea, Marasmiellus virgatocutis var. parvisporus Esteve-Rav. & A.Ortega, Baeospora myosura f. xeruloides A.Ortega & Esteve-Rav.

### Taxa included

**Table:** 

Rank	Scientific Name	Common Name
kingdom	Fungi	
phylum	Basidiomycota	
phylum	Ascomycota	
phylum	Glomeromycota	
order	Agaricales	
order	Aphyllophorales	
order	Dothideales	
order	Glomerales	
order	Lecanorales	
genus	* Bacidia *	
genus	* Baeospora *	
order	Pezizales	
genus	* Bolbitius *	
genus	* Conocybe *	
genus	* Coprinus *	
genus	* Cortinarius *	
genus	* Entoloma *	
genus	* Glomus *	
genus	* Gymnopilus *	
genus	* Gymnopus *	
genus	* Hydropus *	
genus	* Kabatiella *	
genus	* Lecidea *	
genus	* Marasmiellus *	
genus	* Mycena *	
genus	* Naucoria *	
genus	* Rugosomyces *	
genus	* Sarcodon *	
genus	* Trichophaea *	
species	*Bacidia subtilis* Vêzda	
species	*Bolbitius elegans E.Horak, G.Moreno, A.Ortega & Esteve-Rav.	
species	*Coprinus alcobae A.Ortega	
species	*Cortinarius acutopholiotoides Palazón & Mahiques	
species	*Cortinarius assiduus Mahiques, A.Ortega & Bidaud	
species	*Cortinarius aureocistophilus Vila, Contu & Llimona	
species	*Cortinarius ayanamii A.Ortega, Vila, Bidaud & Llimona	
species	*Cortinarius benovairensis Mahiques	
species	*Cortinarius bombycinus Mahiques & Burguete	
species	*Cortinarius castaneoduracinus Chevassut & Rob. Henry	
species	*Cortinarius castaneolens Chevassut & Rob. Henry	
species	*Cortinarius cistohelvelloides Bon	
species	*Cortinarius cistovelatus Vila, A.Ortega & Bidaud	
species	*Cortinarius conico-obtusarum A.Ortega & Chevassut	
species	*Cortinarius contui Rob. Henry & Contu	
species	*Cortinarius crustulinus Malençon	
species	*Cortinarius decipiens (Pers.) Fr.	
species	*Cortinarius diabolicoides Moënne-Locc. & Reumaux	
species	*Cortinarius erythrofuscus Mahiques & A.Ortega	
species	*Cortinarius inusitatus A.Ortega, Bidaud, Suár.-Sant. & Vila	
species	*Cortinarius mahiquesii Vila, A.Ortega & Suár.-Sant.	
species	*Cortinarius murellensis Cors. Gut., Ballarà, Cadiñanos, Palazón & Mahiques	
species	*Cortinarius ortovernus Ballarà & Mahiques	
species	*Cortinarius viscidoamarus A.Ortega & Suár.-Sant.	
species	*Cortinarius xanthosarx Vila, A.Ortega, Bidaud & Suár.-Sant.	
species	*Cortinarius xerophilus Contu & Rob. Henry	
species	*Glomus custos* C. Cano & Dalpé	
species	*Gymnopilus arenophilus A. Ortega & Esteve Rav.	
species	*Gymnopilus maritimus Contu, Guzm.-Dáv., A.Ortega & Vizzini	
species	*Gymnopus pubipes Antonín, A. Ortega & Esteve-Rav.	
species	*Kabatiella bupleuri Bills	
species	*Lecidea circinarioides Casares & Hafellner	
species	*Mycena dunicola M. Villarreal, Esteve-Rav., Barrasa & A.Ortega	
species	*Naucoria decolorata Malençon ex R.Galán, G.Moreno & A.Ortega	
species	*Rugosomyces pudicus* Bon & Contu	
species	*Sarcodon mediterraneus* A.Ortega & Contu	
variety	Conocybe arrhenii var. squamosipes A.Ortega & Esteve-Rav.	
variety	Cortinarius assiduus var. plesiocistus A.Ortega, Vila & Bidaud	
variety	Cortinarius caerulescens var. praetermissus (Bergeron ex Reumaux) A.Ortega & Moënne-Locc.	
variety	Cortinarius caesiostramineus var. cadinanos-aguirrei Moënne-Locc. & A.Ortega	
variety	*Cortinarius croceocaeruleus var. meridionalis Bidaud, A.Ortega & Mahiques	
variety	Cortinarius haasii var. quercus-ilicicola A. Ortega, Suár.-Sant. & J.D. Reyes	
variety	Cortinarius vernus var. nevadavernus Suár.-Sant. & A.Ortega	
variety	Entoloma griseocyaneum var. glyciosmus Esteve-Rav. & A.Ortega	
variety	Gymnopus dryophilus var. lanipes (Malençon & Bertault) A.Ortega, Antonín & Esteve-Rav.	
variety	Hydropus floccipes var. luteipes A.Ortega & M.Zea	
variety	Marasmiellus virgatocutis var. parvisporus Esteve-Rav. & A.Ortega	
variety	*Trichophaea fuscoatra var. punctata Malençon	
form	Baeospora myosura f. xeruloides A.Ortega & Esteve-Rav.	

## Temporal coverage

**Data range:** 1943 10 27 – 2011 5 03.

### Notes

Although the temporal coverage started in 1943, this was a gift and another from 1960 was a single type-specimen included in an exsiccate exchange of lichens. In fact, this collection was started in the mid 1970s and most of the type-specimens were described from 1999 to 2011 (57 type-specimens of 88).

## Collection data

### Collection name

Fungi

### Collection identifier

9b8b659f-8470-4445-926c-8b3f2bc32415

### Parent collection identifier

GDA-GDAC

### Specimen preservation method

Dried

### Curatorial unit

Curatorial unit: 88 with an uncertainty of 0 (specimens); curatorial unit: 49 with an uncertainty of 0 (species); curatorial unit: 19 with an uncertainty of 0 (genera); curatorial unit: 6 with an uncertainty of 0 (order)

## Usage rights

### Use license

Оpen Data Commons Open Database License (ODbL)

## Data resources

### Data package title

Darwin Core Archive Catalogue of type specimens of fungi and lichens deposited in the Herbarium of the University of Granada (Spain)

### Resource link

http://www.gbif.es:8080/ipt/archive.do?r=gda-fungi-tipos

### Alternative identifiers

7ac0504d-0230-4029-afbe-04657ae47c48

### Number of data sets

1

### Data set 1.

#### Data set name

dwca-gda-tipos-fungi

#### Data format

Darwin Core Archive format

#### Number of columns

38

#### Character set

UTF-8

#### Download URL

http://www.gbif.org/dataset/7ac0504d-0230-4029-afbe-04657ae47c48

#### Data format version

1.0

#### Description

This dataset contains the specimen records from the catalogue of the nomenclature types of fungi and lichens in the Herbarium of the University of Granada, Spain (GDA-GDAC). It contains 88 type material of 49 nominal taxa, most from *Agaricales* and the genus *Cortinarius*, described from the western Mediterranean, mainly Spain. For each specimen, locality indication, species name, observation date, collector, type status, related information, associated sequences, other catalogue numbers related to each type, and image URL are recorded. The dataset is associated with an image collection.

**Data set 1.** 

Column label	Column description
dateModified	Date on which the resource was changed.
language	A language of the resource.
institutionCode	The name (or acronym) in use by the institution having custody of the object(s) or information referred to in the record.
colletionCode	The name, acronym, coden, or initialism identifying the collection or data set from which the record was derived.
basisOfRecord	The specific nature of the data record.
occurrenceID	An identifier for the Occurrence (as opposed to a particular digital record of the occurrence). In the absence of a persistent global unique identifier, construct one from a combination of identifiers in the record that will most closely make the occurrenceID globally unique.
catalogNumber	An identifier (preferably unique) for the record within the data set or collection.
occurrenceRemarks	Comments or notes about the Occurrence.
recordedBy	A list (concatenated and separated) of names of people, groups, or organizations responsible for recording the original Occurrence. The primary collector or observer, especially one who applies a personal identifier (recordNumber), should be listed first.
individualCount	The number of individuals represented present at the time of the Occurrence.
otherCatalogNumbers	A list (concatenated and separated) of previous or alternate fully qualified catalog numbers or other human-used identifiers for the same Occurrence, whether in the current or any other data set or collection.
associatedMedia	A list (concatenated and separated) of identifiers (publication, global unique identifier, URI) of media associated with the Occurrence.
associatedSequences	A list (concatenated and separated) of identifiers (publication, global unique identifier, URI) of genetic sequence information associated with the Occurrence.
occurrenceDetails	Comments or notes about the specimen description or identification.
eventDate	The date-time or interval during which an Event occurred. For occurrences, this is the date-time when the event was recorded. Not suitable for a time in a geological context. Recommended best practice is to use an encoding scheme, such as ISO 8601:2004(E).
fieldNotes	One of a) an indicator of the existence of, b) a reference to (publication, URI), or c) the text of notes taken in the field about the Event.
country	The full, unabbreviated name of the country or major political unit in which the organism was collected or observed.
countryCode	Abbreviations on the 2-letters code of the country in which the organism was collected or observed.
stateProvince	The name of the next smaller administrative region than country (state, province, canton, department, region, etc.) in which the Location occurs.
locality	The specific description of the place. Less specific geographic information can be provided in other geographic terms (higherGeography, continent, country, stateProvince, county, municipality, waterBody, island, islandGroup). This term may contain information modified from the original to correct perceived errors or standardize the description.
minimumElevationInMeters	The lower limit of the range of elevation (altitude, usually above sea level), in meters.
maximumElevationInMeters	The upper limit of the range of elevation (altitude, usually above sea level), in meters.
verbatimCoordinates	The verbatim original spatial coordinates of the Location. The coordinate ellipsoid, geodeticDatum, or full Spatial Reference System (SRS) for these coordinates should be stored in verbatimSRS and the coordinate system should be stored in verbatimCoordinateSystem.
verbatimCoordinateSystem	The spatial coordinate system for the verbatimLatitude and verbatimLongitude or the verbatimCoordinates of the Location. Recommended best practice is to use a controlled vocabulary.
decimalLatitude	The geographic latitude (in decimal degrees, using the spatial reference system given in geodeticDatum) of the geographic center of a Location. Positive values are north of the Equator, negative values are south of it. Legal values lie between -90 and 90, inclusive.
decimalLongitude	The geographic longitude (in decimal degrees, using the spatial reference system given in geodeticDatum) of the geographic center of a Location. Positive values are east of the Greenwich Meridian, negative values are west of it. Legal values lie between -180 and 180, inclusive.
coordinateUncertaintyInMeters	The horizontal distance (in meters) from the given decimalLatitude and decimalLongitude describing the smallest circle containing the whole of the Location. Leave the value empty if the uncertainty is unknown, cannot be estimated, or is not applicable (because there are no coordinates). Zero is not a valid value for this term.
identifiedBy	A list (concatenated and separated) of names of people, groups, or organizations who assigned the Taxon to the subject.
dateIdentified	The date on which the subject was identified as representing the Taxon. Recommended best practice is to use an encoding scheme, such as ISO 8601:2004(E).
typeStatus	A list (concatenated and separated) of nomenclatural types (type status, typified scientific name, publication) applied to the subject.
scientificName	The full scientific name, with authorship and date information if known. When forming part of an Identification, this should be the name in lowest level taxonomic rank that can be determined. This term should not contain identification qualifications, which should instead be supplied in the IdentificationQualifier term.
kingdom	The full scientific name of the kingdom in which the taxon is classified.
order	The full scientific name of the order in which the taxon is classified.
genus	The full scientific name of the genus in which the taxon is classified.
specificEpithet	The name of the first or species epithet of the scientificName.
infraspecificEpithet	The name of the lowest or terminal infraspecific epithet of the scientificName, excluding any rank designation.
taxonRank	The taxonomic rank of the most specific name in the scientificName. Recommended best practice is to use a controlled vocabulary.
scientificNameAuthorship	The authorship information for the scientificName formatted according to the conventions of the applicable nomenclaturalCode.

## Additional information

**References cited within the dataset which corresponding to the protologues of the type specimens**

- Ballarà J, et al. (2009) Cortinarius ibero-insulares 2. Fungi Non Delineati, Raro vel Haud Perspecte et Explorate Descripti aut Definite Picti, pars 48-49. Candusso, Alassio, Italia, 1-248.
- Ballarà J, Mahiques R (2009) *Cortinarius ortovernus*, nouvelle espèce printanière de la section *Saturnini*. Journal des Journées européennes du Cortinaire 11: 55-61.
- Bidaud A, Moënne-Loccoz P, Reumaux P (1992) Atlas de Cortinares, Pars IV. Ed. Fédération Mycologique Dauphiné-Savoie, Annecy, France.
- Bills GF, Menéndez VG, Platas G (2012) *Kabatiella bupleuri* sp. nov. (*Dothideales*), a pleomorphic epiphyte and endophyte of the Mediterranean plant *Bupleurum gibraltarium* (*Apiaceae*). Mycologia 104: 962-973. doi: 10.3852/12-003
- Bon M (1992) *Agaricomycetes* mediterraneens ou meridionaux. Documents Mycologiques 22: 51-62.
- Cano C, Bago A, Dalpé Y (2009) *Glomus custos* sp. nov., isolated from a naturally heavy metal-polluted environment in southern Spain. Mycotaxon 109: 499-512. doi: 10.5248/109.499
- Casares-Porcel M, Hafellner J, Gutiérrez-Carretero L (1996) Species of the genus Lecidea (Lecanorales) on gypsum in Spain. Lichenologist 28: 37-47.
- Chevassut G, Henry R (1982) Cortinaires nouveaux ou rares de la region Languedoc Cevennes. Documents Mycologiques 12: 34-65.
- Contu M, Bon M (2000) Une nouvelle espèce de *Rugosomyces* «rougissant». Documents Mycologiques 29: 35-36.
- Esteve-Raventós F, Ortega A (1999) Two news agarics found in Andalusia (Peninsular Spain). Mycotaxon 71: 95-103.
- Esteve-Raventós F, Ortega A (2003) Dos nuevos Tricholomatales ibéricos: *Baeospora myosura* fo. *xeruloides* y Marasmiellus virgatocutis var. parvisporus. Boletín de la Sociedad Micológica de Madrid 27: 63-66.
- Esteve-Raventós F, Villareal M, Barrasa JM, Ortega A (2001) *Mycena dunicola*, a striking new species from the Iberian Peninsula. Mycotaxon 80: 307-313.
- Galán R, Ortega A, Moreno G (1983) Sobre *Naucoria decolorata* G. Malençon (Agaricales). Revista de biologia, Lisboa 12: 61-64.
- Gutiérrez C, Ballarà J, Cadiñanos JA, Palazón F, Mahiques R (2005) *Cortinarius murellensis*, a new Cortinarius subgenus Plegmacium, collected in the course of XXIII J.E.C. Morella-2005. Butlletí Societat Micologia Valenciana 10: 159-160.
- Guzmán-Dávalos L, Ortega A, Contu M, Vizzini A, Rodríguez A, Villalobos-Arámbula AR, Santerre A (2009) *Gymnopilus maritimus* (*Basidiomycota*, *Agaricales*) a new species from coastal psammophilous plant communities of northern Sardinia, Italy, and notes on *G. arenophilus*. Mycological Progress 8: 195-205. doi: 10.1007/s11557-009-0591-7
- Henry R, Contu M (1986) Description d´une nouvelle espece xero-thermophile: *Cortinarius xerophilus* sp. nov. Documents Mycologiques 16: 63-65.
- Horak E, Moreno G, Ortega A, Esteve-Raventós F (2002) *Bolbitius elegans*, a striking new species from southern Spain. Persoonia 17: 615-623.
- Mahiques R (2004) *Cortinarius benovairensis*, nou cortinari de carrascars mediterranis, de la secció obtusi Melot. Butlletí Societat Micologia Valenciana 9: 129-132.
- Mahiques R, Burguete A (2001) *Cortinarius bombycinus*, sp. nov., a new species of subgenus *Telamonia*, section *Sericeocybe*, developped under *Cistus* ssp. in *Quercus ilex* forest. Butlletí Societat Micologia Valenciana 6: 245-248.
- Mahiques R, Ortega A (2002) *Cortinarius erythrofuscus* (subgenus *Telamonia*, section *Firmiores*), a new species from Spain. Persoonia 17: 657-660.
- Mahiques R, Ortega A, Bidaud A (2001) *Cortinarius assiduus* (*Telamonia*, *Firmiores*), nouvelle espèce de la zone méditerranéenne de la Péninsule Ibérique. Bulletin Trimestriel de la Fédération Mycologique Dauphiné-Savoie 162: 41-47.
- Malençon G, Bertault R (1970) Flore des champignons superieurs du Maroc. Tome I, Rabat.
- Malençon G, Llimona X (1980) Champignons de la Péninsule Ibérique VI. Est et sud-est. Anales de Biología, Universidad de Murcia 34: 47-135.
- Moënne-Loccoz P, Reumaux P (1990) Atlas des Cortinaires, Pars II. Ed. Fédération Mycologique Dauphiné-Savoie, Annecy, France.
- Ortega A, Antonín V, Esteve-Raventós F (2003) Three interesting thermophilic taxa of *Gymnopus* (*Basidiomycetes*, *Tricholomataceae*): *G. pubipes* sp. nov., G. pubipes var. pallidopileatus var. nov. and G. dryophilus var. lanipes comb. nov. Mycotaxon 85: 67-75.
- Ortega A, Bidaud A, Mahiques R (1997) Contribución al estudio del género *Cortinarius* en España peninsular. II Parte. Cryptogamie Mycologie 18: 227-231.
- Ortega A, Chevassut G (1999) *Cortinarius conico-obtusarum* (*Telamonia*), a new species from southern Spain. Documents Mycologiques 29: 79-81.
- Ortega A, Contu M (1991) Una nuova specie di *Sarcodon* (*Aphyllophorales*, *Telephoraceae*) dalle comunita arenicole mediterranee. Boletín de la Sociedad Micológica de Madrid 15: 149-152.
- Ortega A, Esteve-Raventós F (1998) Contribution to the study of the mycoflora of Andalusia (Spain) XIII. *Agaricales* VI. Taxonomic notes on two interesting Agarics from the Iberian Peninsula. Nova Hedwigia 67: 107-113.
- Ortega A, Esteve-Raventós F (2003) New and interesting species of *Coprinus* (*Coprinaceae*, *Agaricales*) from Andalusia (Southern Spain). Nova Hedwigia 76: 465-475. doi: 10.1127/0029-5035/2003/0076-0465
- Ortega A, Esteve-Raventós F (2005) A new species of Gymnopilus (Cortinariaceae) from sandy soils in *Pinus* forests. Persoonia 18: 505-510.
- Ortega A, Mahiques R (2002) Study of some species of the genus *Cortinarius*, section *caerulescens* (R. Henry) ex Moënne-Loccoz & Reumaux in peninsular Spain. Mycotaxon 83: 435-445.
- Ortega A, Moënne-Loccoz P (2003) *Cortinarius praetermissus* Bergeron ex Reumaux (section *caerulescentes* (R. Henry) ex Moënne-Loccoz et Remaux). Documents Mycologiques 32: 37-41.
- Ortega A, Suárez-Santiago VN, Reyes JD (2008) Morphological and ITS identification of *Cortinarius* species (section *Calochroi*) collected in Mediterranean Quercus woodlands. Fungal Diversity 29: 73-88.
- Ortega A, Suárez-Santiago VN, Vila J (2009) Two new species of *Cortinarius* collected under *Quercus rotundifolia* in the Mediterranean area of southern Spain. Fungal Diversity 36: 89-99.
- Ortega A, Vila J, Bidaud A, Llimona X (2000) *Cortinarius ayanamii* A.Ortega, Vila, Bidaud & Llimona. Cortinaire cistophile nouveau, trouvé en Catalogne. Bulletin Trimestriel de la Fédération Mycologique Dauphiné-Savoie 157: 23-26.
- Ortega A, Vila J, Bidaud A, Mahiques R, Contu M (2007) Notes on four mediterranean *Cortinarius* fruiting in sclerophilous and heliophilous plant ecosystems. Mycotaxon 101: 137-147.
- Ortega A, Zea M (1991) Hydropus floccipes var. luteipes Ortega & Zea var. nov., en España meridional. Boletín de la Sociedad Micológica de Madrid 15: 189-191.
- Palazón F, Mahiques R (2007) *Cortinarius acutopholiotoides*, sp. nov., nuevo cortinario de la Sección *Hydrocybe*, localizado bajo *Quercus* en Zamora, España. Journal des Journées européennes du Cortinaire 9: 76-83.
- Suárez-Santiago VN, Ortega A, Peintner U, López-Flores I (2009) Study on Cortinarius subgenus Telamonia section Hydrocybe in Europe, with especial emphasis on Mediterranean taxa. Mycological Research 113: 1070-1090. doi: 10.1016/j.mycres.2009.07.006
- Vêzda A (1961) Lichenes novi vel rariores Sudetorum occidentalium. Preslia 33: 365-368.
- Vila J, Llimona X (2006) Novesdades sobre el component fúngic de les comunitats de *Cistus* de Catalunya. II. Revista Catalana de Micología 28: 167-207.
- Vila J, Ortega A, Bidaud A (2007) Deux cortinaires remarquables de la Péninsule Ibérique. Bulleti Société mycologique de France 123: 221-232.
- Vila J, Ortega A, Suárez-Santiago VN, Llimona X (2008) *Cortinarius mahiquesii*, a new subhypogeous species from Catalonia (Iberian Peninsula). Persoonia 21: 153-157. doi: 10.3767/003158508X388380

## Supplementary Material

Supplementary material 1 GDA_type specimens_fungi Data type: tables correspond to figures
Brief description: This file contained a data set of the type-specimens occurrences, and numbers and tables corresponding to the figures in the paper.
File: oo_42773.xlsx Vizoso, M.T.
